# Structure-Property Relationships in Graphene-Based Strain and Pressure Sensors for Potential Artificial Intelligence Applications

**DOI:** 10.3390/s19051250

**Published:** 2019-03-12

**Authors:** Zewei Luo, Xiaotong Hu, Xiyue Tian, Chen Luo, Hejun Xu, Quanling Li, Qianhao Li, Jian Zhang, Fei Qiao, Xing Wu, V. E. Borisenko, Junhao Chu

**Affiliations:** 1 Shanghai Key Laboratory of Multidimensional Information Processing, Department of Electronic Engineering, East China Normal University, Shanghai 200241, China; 52181213021@stu.ecnu.edu.cn (Z.L.); 10172100328@stu.ecnu.edu.cn (X.H.); 51181213032@stu.ecnu.edu.cn (X.T.); 52161213022@stu.ecnu.edu.cn (C.L.); 52151213012@stu.ecnu.edu.cn (H.X.); 51161213021@stu.ecnu.edu.cn (Q.L.); 51171213058@stu.ecnu.edu.cn (Q.L.); jzhang@ee.ecnu.edu.cn (J.Z.); jhchu@mail.sitp.ac.cn (J.C.); 2 Shanghai Institute of Intelligent Electronics & Systems, Fudan University, Shanghai 200433, China; 3 Department of Electronic Engineering, Tsinghua University, 30 Shuangqing Road, Beijing 100084, China; 4 Belarusian State University of Informatics and Radioelectronics, P. Browka 6, 220013 Minsk, Belarus; borisenko@bsuir.by; 5 National Research Nuclear University MEPhI (Moscow Engineering Physics Institute), Kashirskoe Shosse 31, 115409 Moscow, Russia

**Keywords:** graphene, strain sensor, pressure sensor, structure-property, artificial intelligence

## Abstract

Wearable electronic sensing devices are deemed to be a crucial technology of smart personal electronics. Strain and pressure sensors, one of the most popular research directions in recent years, are the key components of smart and flexible electronics. Graphene, as an advanced nanomaterial, exerts pre-eminent characteristics including high electrical conductivity, excellent mechanical properties, and flexibility. The above advantages of graphene provide great potential for applications in mechatronics, robotics, automation, human-machine interaction, etc.: graphene with diverse structures and leverages, strain and pressure sensors with new functionalities. Herein, the recent progress in graphene-based strain and pressure sensors is presented. The sensing materials are classified into four structures including 0D fullerene, 1D fiber, 2D film, and 3D porous structures. Different structures of graphene-based strain and pressure sensors provide various properties and multifunctions in crucial parameters such as sensitivity, linearity, and hysteresis. The recent and potential applications for graphene-based sensors are also discussed, especially in the field of human motion detection. Finally, the perspectives of graphene-based strain and pressure sensors used in human motion detection combined with artificial intelligence are surveyed. Challenges such as the biocompatibility, integration, and additivity of the sensors are discussed as well.

## 1. Introduction

Wearable devices can be worn directly on the user, embedded in clothing, or implanted in the body to detect human health motion [1,2,3,4,5]. These flexible sensors can monitor physiological parameters such as pulse, blood pressure, body temperature, and heart rate signals of the human body with high efficiency and subtle discomfort, as a part of advanced devices [6,7,8,9,10,11,12].

Wearable sensors possess a wide range of applications in wearable devices due to their light weight, good ductility, flexibility, and suitability for large-area manufacturing processes [13,14,15]. However, in order to mount easier the wearable sensors on the human skin for real-time human motion detection, several performance and parameter requirements need to be fulfilled. Lightweight, flexibility, stretchability, durability, biocompatibility, and low power consumption are crucial properties for wearable sensors [16,17,18].

In general, researchers take advantage of the superior electrical, chemical, and optical properties of nanomaterials for signal sensing, combining mechanical, flexibility, stretchability, and durability of polymers to make the sensors flexible. These novel sensors are mainly strain sensors [19,20,21] and pressure sensors [22,23]. Nowadays, various wearable sensors have been widely developed in health monitoring, human motion detection, device system integration, human-machine interactions, and artificial intelligence [24,25,26,27,28].

**Figure 1:**
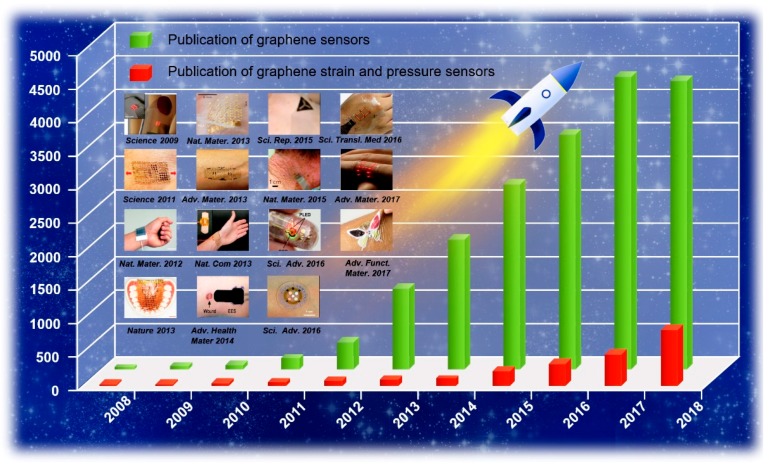
Trends in the development of graphene-based sensors and graphene-based strain and pressure sensors. The data is from the Web of Science. Hundreds of papers have been published, and a selected subset is represented since 2008 [36,37,38,39,40,41,42,43,44,45,46,47,48,49,50,51,52]. Copyright 2009, American Association for the Advancement of Science; Copyright 2011, American Association for the Advancement of Science; Copyright 2008, Wiley; Copyright 2012, Nature Publishing Group; Copyright 2013, Nature Publishing Group; Copyright 2013, Nature Publishing Group; Copyright 2013, Wiley; Copyright 2013, Nature Publishing Group; Copyright 2014, Wiley; Copyright 2015, Nature Publishing Group; Copyright 2015, Wiley; Copyright 2016, American Association for the Advancement of Science; Copyright 2016, American Association for the Advancement of Science; Copyright 2016, American Association for the Advancement of Science; Copyright 2017, Wiley; Copyright 2017, Wiley.

Graphene demonstrates outstanding characteristics such as perfect mechanical strength, good electrical properties, chemical stability, and high thermal conductivity [29,30] which are all required for the sensing materials of wearable mechanical sensors. Due to ultrahigh sensitivity, graphene is one of the best nanomaterials for pressure and strain sensing applications [31,32]. It is critical and necessary to further study and develop graphene-based strain and pressure sensors. As shown in Figure 1, graphene-based strain and pressure sensors have achieved conspicuous progress. Over the last decade, graphene-based sensors have been categorized into three conversion mechanisms such as resistive, capacitance, and piezoelectricity, showing a vigorous development trend [33,34,35].

**Figure 2:**
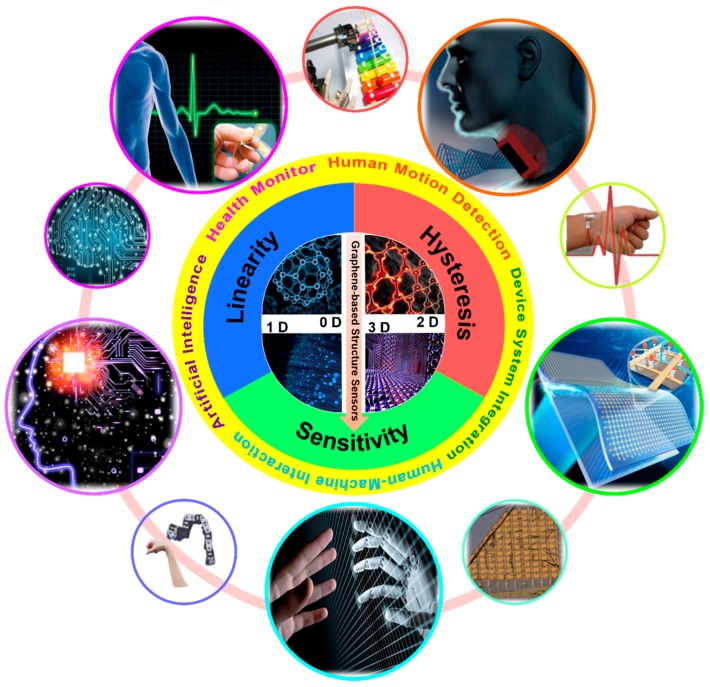
Overview of the graphene-based strain and pressure sensors in the various fabrications of the structures and wide range of potential applications [76,77,78,79]. Copyright 2015, Wiley; Copyright 2014, Nature Publishing Group; Copyright 2016, Nature Publishing Group; Copyright 2017, Nature Publishing Group; Copyright Google Images.

In this review, we place particular emphasis on structure-property and the potential applications of the latest graphene-based strain and pressure sensors. The fundamentals and transduction mechanisms of the graphene-based strain and pressure sensors are described. As shown in Figure 2, we highlight the latest progress and breakthrough in graphene-based strain and pressure sensors classified in four structures including 0-dimensional (0D) fullerene [53,54], 1-dimensional (1D) fiber [55,56,57], 2-dimensional (2D) film [58,59,60,61], and 3-dimensional (3D) porous structure [62,63,64,65] to guarantee various properties. Herein, the 0D structure graphene-based sensors are defined as the sensors that use a 0D composite structure in the sensing layer. The 1D structure graphene-based sensors are defined as sensors that use fibers like composite structures in the sensing layer. The 2D structure focuses on sensors that use 2D planar film. The 3D structure refers to a porous structure. Graphene is used as the main sensing material in the above structures. The above “composite structure” means that there are other materials in the sensing layer except graphene [66,67]. Additionally, major technical parameters in graphene-based strain and pressure sensors such as linearity, sensitivity, and hysteresis properties for accurate sensing are described. Graphene-based strain and pressure sensors show various applications including device system integration, health monitoring, human motion detection, human–machine interaction, and artificial intelligence [68,69]. Finally, we summarize recent development trends and application forecasts, especially the challenges in combination with artificial intelligence [70,71,72,73,74,75].

## 2. Fundamentals of Graphene-Based Strain and Pressure Sensors

### 2.1. Classification of Graphene-Based Strain and Pressure Sensors

**Figure 3:**
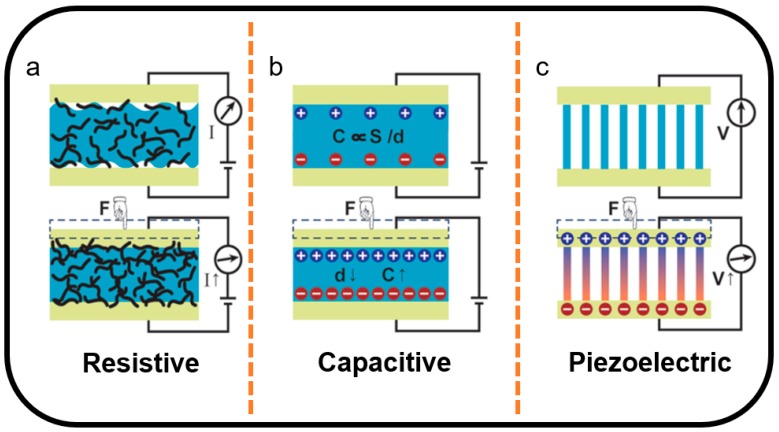
Schematic illustrations of the transduction methods: (**a**) resistive, (**b**) capacitive, and (**c**) piezoelectric [79,96,105,106]. Copyright 2015, American Association for the Advancement of Science.

The excellent characteristics and the efficient productive ability of graphene make it a suitable choice for the sensing materials of strain and pressure sensors. Graphene-based strain and pressure sensors exhibit one of the highest sensitivities and gauge factor [80,81,82]. During the last few years, researchers have made great progress in graphene-based strain and pressure sensors [83]. In graphene-based strain and pressure sensors, graphene [84,85,86,87] is familiarly used as active material to sense physical signal including strain and pressure [88,89]. Due to the excellent electrical conductivity, graphene materials are frequently used as a conducting layer or electrodes of graphene-based strain and pressure sensors. Furthermore, various graphene structures such as 0D fullerene, 1D fiber, 2D film, and 3D porous structure guarantee the multifunction of graphene-based strain and pressure sensors for applications in different scenarios. As shown in Figure 3, traditional transduction methods of graphene-based strain and pressure sensors include resistive [90,91], capacitance [92,93], and piezoelectricity [94,95]. The details of these transduction methods are presented in this section.

Resistive sensors convert external forces into a variation of resistance, which can be directly detected by a pre-built detection circuit through changes in the electrical signals. It obtains a resistive sensing signal through the change of the resistance [96]. Due to a simple measurement method and the large scope of applications [97], resistive sensors have been widely used. As shown in Figure 3a, the resistive effect is generated by an applied external force changing the conductive path of the sensing material, which changes the resistance [98,99,100]. The resistive effect is an inherent characteristic of graphene which makes it a desired sensing material for strain and pressure sensors. The high conductivity and favorable mechanical properties of graphene enable the graphene-based resistive sensor to have ultrahigh sensitivity [101]. As a common type of strain and pressure sensor, the advantages of graphene-based resistive sensors are a wide detection range, simple equipment construction, and signal testing [84,102,103,104]. Due to these advantages, graphene-based resistive sensors have attracted great attention. Moreover, graphene-based resistive sensors also demonstrate unlimited potential in frontier applications such as human motion detection and artificial intelligence.

The capacitive sensor is another traditional type of graphene-based strain and pressure sensor [107,108]. Capacitive sensors can detect different forms of force by converting mechanical stimulus signals into displacement signals [109,110]. The change of displacement causes a change in capacitance. As shown in Figure 3b, capacitive sensors detect force variation in different directions by changing the effective area of the sensing material and the spacing of the parallel plates to obtain an electrical signal [101]. The sensitivity and stability of capacitive sensors mainly depend on the favorable compression properties of the dielectric layer. Due to the excellent conductivity, favorable mechanical properties, and large specific surface area, graphene is the ideal electrical conductor and electrode for capacitive sensors [111]. The extreme sensitivity of a capacitive sensor to weak changes makes it widely used in the detection of static or tiny forces [112,113,114,115].

Piezoelectric materials are special materials which can generate electrical charges under mechanical stress. The piezoelectric effect is caused by the presence of an oriented non-centrosymmetric crystal structure in the piezoelectric material, resulting in an electric dipole moment [116]. The higher the piezoelectric coefficient of a piezoelectric material, the higher the energy conversion efficiency it has [117]. Therefore, highly sensitive and fast-responding piezoelectric materials are widely used in pressure sensors that convert pressure into electrical signals. Previous research has shown that single-layer graphene can achieve a negative piezoelectric effect, and two-layer and multi-layer graphene can achieve a positive piezoelectric conductance effect [118,119]. Graphene-based piezoelectric sensors have been used to detect continuous static pressure signals and perpendicular vibrations due to their ultrafast response time and ultrahigh sensitivity [120,121].

### 2.2. Major Parameters of Graphene-Based Strain and Pressure Sensors

Different fabrication procedures and structures (0D fullerene, 1D fiber, 2D film, 3D porous structure) in graphene-based strain and pressure sensors result in various properties and functions for applications. It is crucial to list and classify the parameters of graphene-based strain and pressure sensors. These parameters can be used to visually distinguish the characteristics and functions of the sensors. For graphene-based strain and pressure sensors, there are several major parameters including sensitivity, gauge factor (mostly for the strain sensor), detection range, linearity, hysteresis, response time, and relaxation time. It is worth noting that sensitivity exists in pressure sensors while the gauge factor exists in the strain sensors.

The sensitivity of pressure sensors generally refers to the ratio between the variables involved in the output and input signals. For graphene-based pressure sensors with different transduction mechanisms, the input and output signals are different. For instance, the sensitivity of resistive pressure sensors is calculated by dividing the relevant variation of resistance by the variation of the applied force, as shown in Formula (1). In the same way, sensitivities of capacitive and piezoelectric pressure sensors correspond to capacitance and voltage, respectively.

(1) $$Sensitivity=\left|\frac{\Delta R/R_{0}}{\Delta F}\right|$$

Gauge factor, which makes no sense to pressure sensors, is an important parameter for strain sensors. Gauge factor (GF), also named strain factor, of a strain sensor is the ratio of the relevant change in electrical resistance R, to the mechanical strain ε, which means this parameter is only significant for the resistive strain sensor. (Formula (2))

(2) $$GF=\left|\frac{\Delta R/R_{0}}{\varepsilon }\right|$$

Linearity is an important indicator to describe the static characteristics of a sensor. It is used to characterize the parameters whose actual characteristics do not match the fitted line. In certain conditions, the ratio of the maximum deviation between the sensor calibration curve to the fitted line and the full-scale output is called linearity, also known as nonlinearity error [122]. For the graphene-based strain and pressure sensors, it is still a technical challenge to balance the relationship between sensitivity and linearity [123,124]. At present, researchers still cannot achieve both high sensitivity and good linearity for graphene-based strain and pressure sensors, which needs further study [125,126,127].

The phenomenon that the input and output characteristic curves do not coincide during the input quantity changes from small to large and from large to small is called hysteresis. It refers to the degree of inconsistency between the forward stroke characteristics and the reverse stroke characteristics of the sensor under the same operating conditions [128,129]. Hysteresis is another important indicator of sensor performance. Therefore, various factors affecting hysteresis must be strictly controlled in the production process of the sensor. For the graphene-based strain and pressure sensors, when the sensor is stretched or compressed and released, graphene flakes need several seconds or milliseconds to return to their original position, by which occurs hysteresis [130,131]. High hysteresis reduces the durability and robustness of the sensor. Hence hysteresis is an important parameter for graphene-based strain and pressure sensors.

The detection range of strain and pressure sensors is the maximum and minimum values that can be accurately measured. Mechanical sensors are commonly used to measure tiny disturbances, especially in biomedical applications. Therefore, the detection range is also an important parameter to use to judge whether the measurement of the sensor is effective.

Response time and relaxation time are two parameters to describe the speed of the response of the sensor at the loading and unloading process, respectively. Response time and relaxation time are important technical indicators of the sensor, reflecting the response speed of the sensor to the signal; the smaller the value, the faster the response. The response time determines the frequency of the signal sampling. Thus, it is crucial for the sensor.

Major parameters for recent graphene-based strain and pressure sensor research are summarized and classified in Table 1 and Table 2, respectively. The “Sensing Materials” column identifies the materials used in the sensing layer of the sensors. The “Transduction Mechanisms” column describes the transduction mechanisms and structure of the sensors [108,132,133]. The other columns are for the major parameters of the sensors [134,135].

## 3. A Graphene-Based Inorganic Pressure Sensor in Various Dimensionalities

### 3.1. Zero Dimensional

**Figure 4:**
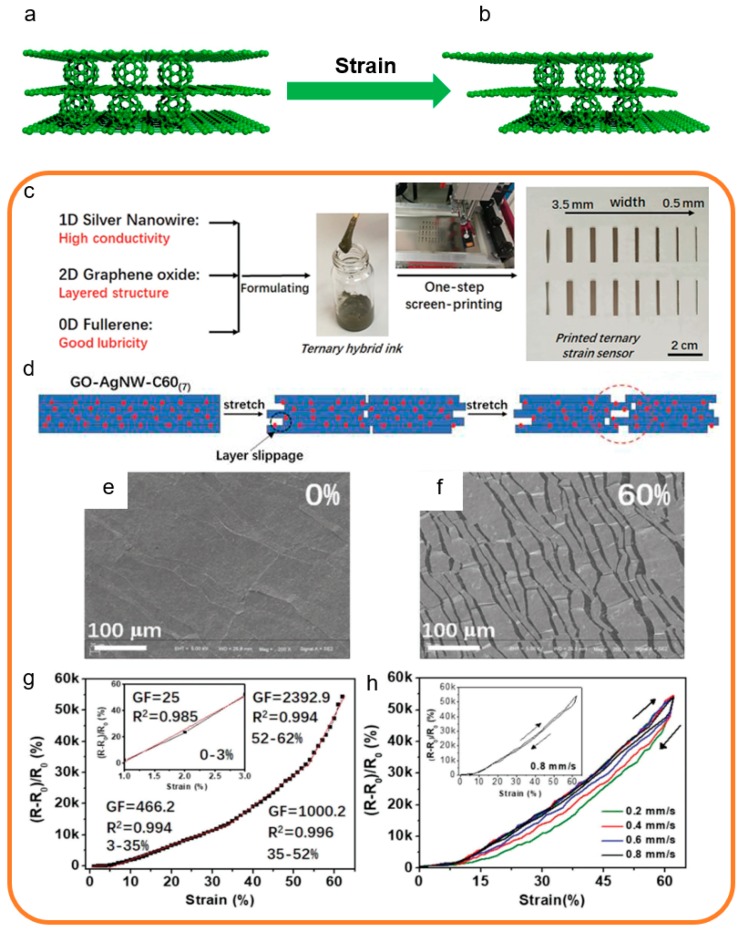
Fabrication processes and structural characterization of 0D structure strain sensors [136]. Copyright 2018, Wiley. (**a**) The structure diagram for 0D strain sensors based on 0D fullerene structure before stretch. (**b**) The structure diagram of the 0D strain based on 0D fullerene structure after stretch. (**c**) The preparation process of this 0D structure strain sensor. (**d**) Schematic illustration of a sensing mechanism for films under stretching. (**e**) Surface SEM images for sensing films at 0% applied strains. (**f**) Surface SEM images for sensing films at 60% applied strains. (**g**) The Gauge factor (GF) and linear behavior of the strain sensor. (**h**) The hysteresis of the strain sensor.

Herein, the 0D structure graphene-based sensors are defined as the sensors that use a 0D composite structure in the sensing layer, and 0D fullerene as a lubricant and graphene as the main sensing material. The structural characterizations of 0D structure strain and pressure sensors are shown in Figure 4a,b. The 0D fullerene structure is incompact before the strain and pressure as shown in Figure 4a. In the loading state, the 0D fullerene structure becomes compact (Figure 4b).

Chen et al. proposed a 0D structure graphene-based strain sensor which has brilliant properties including good linearity, high sensitivity, and low hysteresis [136]. As shown in Figure 4c, this 0D structure strain sensor was assembled by screen-printing from a ternary aqueous ink on the stretchable substrate. Figure 4d shows the sensing mechanism of this 0D structure strain sensor. The stretching in the sensing layer undergoes slippage between sensing material layers. Due to this sensor being fabricated from ternary composites, it is worth researching what role the 0D structure plays in the sensor. As shown in Figure 4e,f, applying different degrees of stress to the film leads to microcrack formation on the surface of the film and spreads evenly, which demonstrates the impact of the 0D structure on the sensing mechanism. It serves as a lubricant to reduce friction between adjacent layered materials in the sensor. In addition, this 0D structure strain sensor exhibits both large stretchability and ultrahigh gauge factor. As shown in Figure 4g, at up to 62% strain, the gauge factor of this sensor reaches 2392.9. This 0D structure strain sensor also exhibits negligible hysteresis at 0.8 mm s^−1^ strain rate, as shown in Figure 4h.

Due to the complicated preparation process and limited role in sensing materials, there are rare studies on the 0D structure graphene-based strain and pressure sensors. It is difficult to support a complete sensing structure alone for 0D structure graphene, which is usually used as a lubricant to improve sensor performance. Most 0D structure graphene-based strain and pressure sensors have negligible hysteresis and ultrahigh sensitivity due to the structural lubrication of the 0D structure. However, its linearity still needs to be improved.

### 3.2. One Dimensional

**Figure 5:**
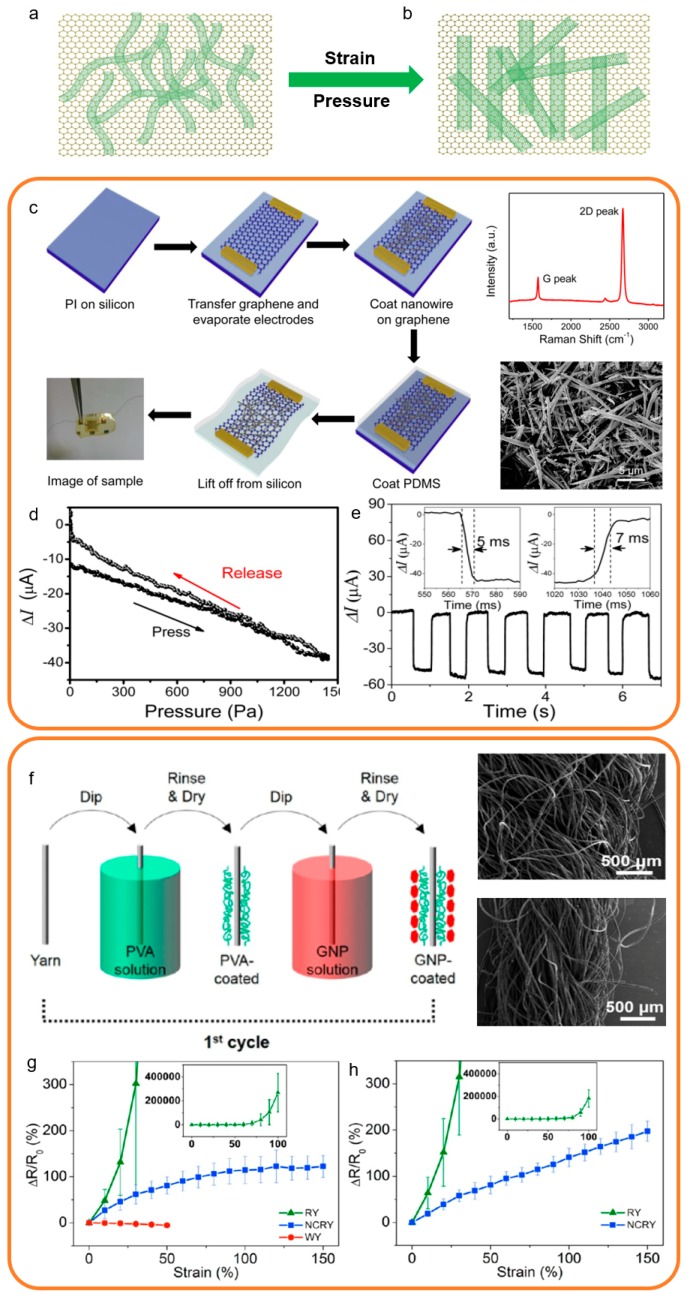
Fabrication processes and structural characterization of 1D structure strain and pressure sensors [34,163]. Copyright 2017, American Chemical Society; Copyright 2015, American Chemical Society. (**a**) The structure diagram of the 1D strain and pressure sensors based on 1D graphene structure before the strain and pressure. (**b**) The structure diagram of the 1D strain and pressure sensors based on 1D graphene structure after the strain and pressure. (**c**) The fabrication process of a 1D structure pressure sensor and its representations. (**d**) The sensitivity and linearity of this pressure sensor. (**e**) The hysteresis of this pressure sensor. (**f**) The fabrication process of a 1D structure strain sensor and its representations. (**g**) The sensitivity and linearity of three types of strain sensors. (**h**) The function of the Polydimethylsiloxane (PDMS) coating.

Herein, the 1D structure graphene-based sensors are defined as sensors that use fibers like composite structure in the sensing layer, and graphene as the main sensing material. In general, to fabricate the 1D structure, the polymer material is used as a substrate and the graphene is deposited on the polymer by chemical or physical methods. The representative structural characterizations of 1D structure strain and pressure sensors are shown in Figure 5a,b. The 1D fibers are tortuous before the strain and pressure as shown in Figure 5a. In the loading state, the 1D fiber structure becomes unbent (Figure 5b) [157,158,159,160,161,162].

In a typical case, Xu et al. described a flexible graphene-based pressure sensor by using a novel material called PbTiO_3_ nanowires (PTNWs), which has been applied in human motion detection and health monitoring [34]. Compared to the traditional chemical vapor deposition (CVD)-grown graphene-based pressure sensor, this graphene-based pressure sensor shows a higher sensitivity. The fabricating processes of this 1D structure graphene pressure sensor are shown in Figure 5c. A Raman spectrum of graphene shows a small D peak at 1350 cm^−1^, which depicts low-density defects or disordered carbon in graphene. The distributive diameter of the PTNWs is about 500 nm, with the lengths reaching up to 10 μm. This sensor takes advantage of the polarization charges in PbTiO_3_ nanowires to stimulate carrier mobility of the graphene, which drastically increases the sensitivity. As shown in Figure 5d, this sensor exhibits both good linearity ranging from 0 to 1400 Pa and ultrahigh sensitivity up to 9.4 × 10^−3^ kPa^−1^. The response time and relaxation time are 5 ms and 7 ms, respectively, as shown in Figure 5e, which indicates negligible hysteresis of this sensor.

In the other representative case, Oopark et al. proposed different types of graphene-based strain sensors possessing large stretchability, high sensitivity, and special negative sensing response, which can also be used in human motion detection and health monitoring [163]. As shown in Figure 5f, these graphene-based fiber strain sensors were fabricated from a graphene nanoplatelet dispersion and a poly vinyl alcohol solution using the layer-by-layer assembly technique. The SEM images of the graphene-based strain sensors without strain and with strain demonstrate the characterization of the 1D structure, which determines the properties of the graphene-based strain sensors. Figure 5g,h show the wide detection scale up to 150%, excellent linearity stretching up to 100%, high sensitivity, and negligible hysteresis of these sensors. Especially, the wool yarn graphene-based strain sensor demonstrates peculiar negative resistive property.

The 1D structure is a common form of graphene-based strain and sensors, which can be used in most traditional transduction methods. However, the dimensional limitations cause anisotropy limitations. In general, the 1D structure graphene-based strain and sensors can only be pressured or stretched in one direction. Most 1D structure graphene-based strain and pressure sensors have negligible hysteresis and favorable linearity. However, the detection scale of the 1D structure sensors is limited and the sensitivity is relatively tiny compared to other structures.

### 3.3. Two Dimensional

**Figure 6:**
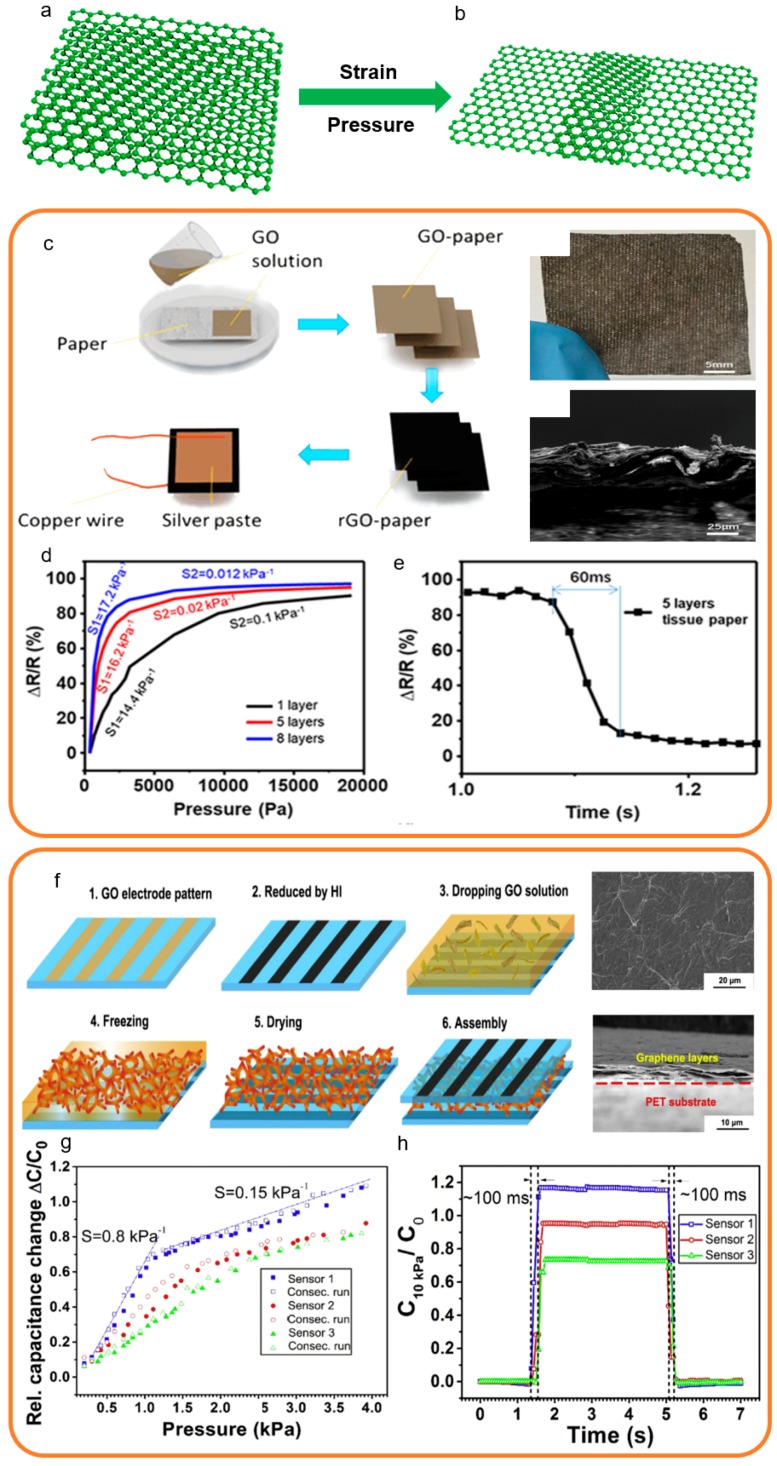
Fabrication processes and structural characterization of 2D structure strain and pressure sensors [111,155]. Copyright 2017, Elsevier; Copyright 2017, American Chemical Society. (**a**) The structure diagram of the 2D strain and pressure sensors based on 2D graphene layers structure before the strain and pressure. (**b**) The structure diagram of the 2D strain and pressure sensors based on 2D graphene layers structure after the strain and pressure. (**c**) The fabrication process of a 2D structure pressure sensor and its representations. (**d**) The sensitivity and linearity of this pressure sensor. (**e**) The response time and hysteresis of this pressure sensor. (**f**) The fabrication process of a 2D structure pressure sensor and its representations. (**g**) The sensitivity and linearity of this pressure sensor (**h**) The response time and hysteresis of this pressure sensor.

Herein, we present the 2D structure focus on sensors that use 2D planar film and graphene as the main sensing material. The 2D structure can be obtained by methods such as suction filtration, CVD growth, chemical synthesis, etc. The classical structural characterization of 2D structure strain and pressure sensors is shown in Figure 6a,b. The 2D graphene layer structure is fluffy before the strain and pressure as shown in Figure 6a. In the loading state, the 2D graphene layer structure becomes impacted (Figure 6b) [164,165,166,167,168].

In a typical case, Ren et al. proposed a paper of the 2D graphene-based pressure sensor which has wide potential in the use of human motion detection and health monitoring [155]. This 2D graphene-based pressure sensor has ultrahigh sensitivity, stable repeatability, and good hysteresis. As shown in Figure 6c, this 2D graphene-based strain sensor was fabricated by several simple steps. The graphene 2D structure of this paper like the 2D graphene-based pressure sensor can be clearly observed in the optical image of graphene paper. The optical image of the cross-section shows the folds and collapses in the graphene film, which makes it so called graphene paper. The sensing mechanism of this paper like the 2D graphene-based pressure sensor is the many voids and pores between the graphene layers. When pressure is applied, the indirect contact dots of the graphene sheets rapidly increase, and the resistance rapidly decreases, which is the main reason why this sensor has ultrahigh sensitivity up to 17.2 kPa^−1^ in the range of 0–2 kPa (Figure 6d). Figure 6e shows the response time is about 60 ms which indicates negligible hysteresis of this sensor.

In the other representative case, a high sensitivity and negligible hysteresis capacitive graphene-based pressure sensor is proposed by Sun et al. which can be used in human-machine interactions and artificial intelligence [111]. As shown in Figure 6f, this 2D graphene-based strain sensor is fabricated by individual reduced graphene oxide materials in six steps. The SEM images apparently indicate the 2D graphene layers structure in sensing materials and substrates and the graphene electrodes demonstrate homogeneity and smoothness. By changing the density of the sensing material, the detection range of this 2D graphene-based strain sensor can be changed. In Figure 6g, it is shown that this 2D graphene-based strain sensor has ultrahigh sensitivity up to 0.8 kPa^−1^ at extreme low-pressure regime about 0–1 kPa. This sensor also exhibits fast response time about 100 ms as shown in Figure 6h.

The 2D structure is the most hackneyed form of graphene-based strain and sensors, which can be applied in all transduction methods. The 2D structure graphene-based strain and pressure sensors can be pressured or stretched in all directions in the plane. According to different needs, the 2D structure graphene-based strain and pressure sensors can detect large or tiny forces. Most 2D structures have negligible hysteresis, favorable linearity, and above-average sensitivity. Even if the 2D structure graphene-based strain and sensors have the above benign properties, it is impossible to generate a large deformation by a tiny force because the sensing layer is essentially a thin film.

### 3.4. Three Dimensional

**Figure 7:**
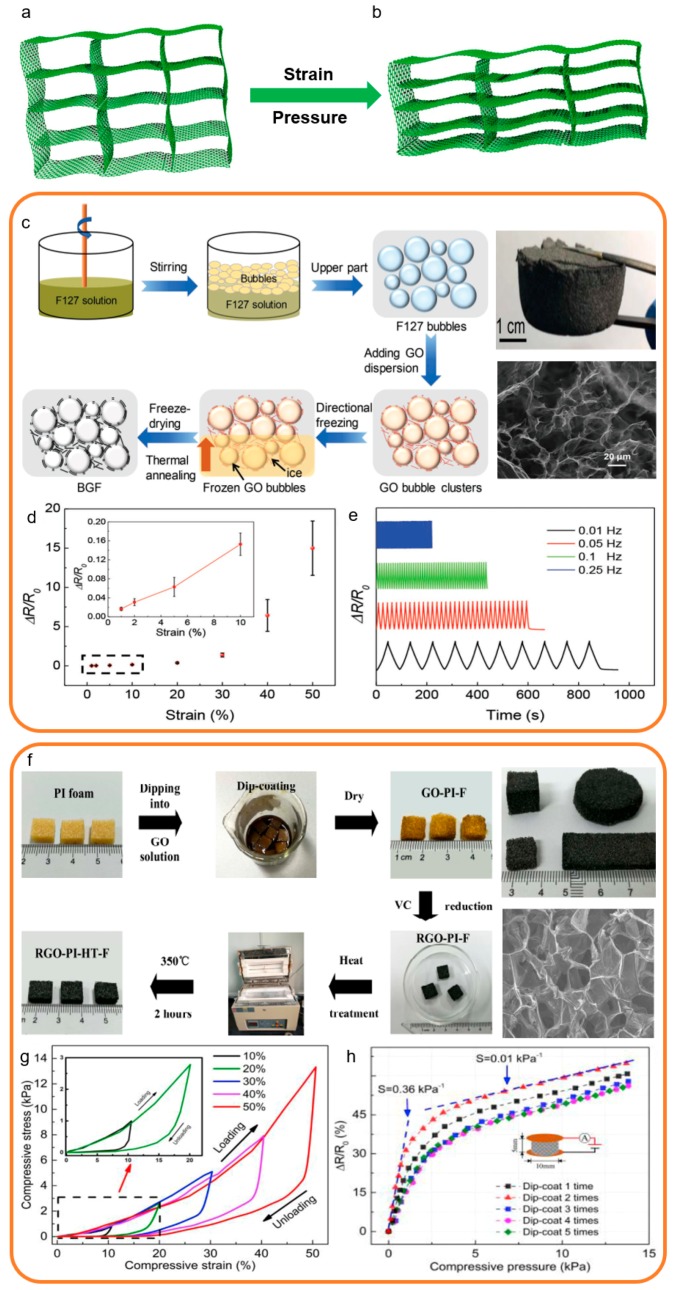
Fabrication processes and structural characterization of 3D structure strain and pressure sensors [142,153]. Copyright 2017, Wiley; Copyright 2018, Elsevier. (**a**) The structure diagram of the 3D strain and pressure sensors based on 3D graphene sponge structure before the strain and pressure. (**b**) The structure diagram of the 3D strain and pressure sensors based on 3D graphene sponge structure after the strain and pressure. (**c**) The fabrication process of a 3D structure strain and pressure sensor and its representations. (**d**) The sensitivity and linearity of this strain and pressure sensor. (**e**) The repeatability and hysteresis of this strain and pressure sensor. (**f**) The fabrication process of a 3D structure strain and pressure sensor and its representations. (**g**) The linearity and hysteresis of this strain and pressure sensor. (**h**) The sensitivity and hysteresis of this pressure sensor.

Herein, the 3D structure refers to the porous structure and graphene as the main sensing material. The methods for preparing the 3D structure graphene-based strain and pressure sensors are various, such as skeleton erosion and freeze drying. In general, the 3D graphene structure has an internal loose porous structure, which makes it more compressible. The typical structural characterizations of 3D structure strain and pressure sensors are shown in Figure 7a,b. The 3D graphene sponge structure is loose before the strain and pressure, as shown in Figure 7a. In the loading state, the 3D graphene sponge structure becomes shriveled (Figure 7b) [169,170,171,172].

Recently, a 3D structure graphene-based strain and pressure sensor was prepared by Zhu et al. by using a neoteric method [142]. This sensor is fabricated by a novel material named as the 3D bubble-derived graphene-based porous material which shows ultrahigh sensitivity, magnificent linearity, and great hysteresis. The above superiorities make it suitable for use in vibration testing and health monitoring. The preparation process of bubble-derived graphene foams (BGFs) was demonstrated in Figure 7c, including the bubbling and ice templating steps. The optic image shows the in-kind shooting of the BGFs. The SEM image shows the porous structure inside the BGFs. As shown in Figure 7d, this 3D structure graphene-based strain and pressure sensor exhibits good linearity over different strain ranges. This phenomenon is due to the different degrees of tearing and fracture under stresses in the internal structure of three-dimensional graphene. This 3D structure graphene-based strain and pressure sensor is also able to test subtle vibration, as shown in Figure 7e.

In another representative case, a novel graphene-based strain and pressure sensor was prepared by dip-coating a polyimide foam template followed by chemical reduction and thermal reduction [153]. This 3D structure graphene-based strain and pressure sensor displays high sensitivity and good linearity, which can be used in health monitoring and human motion detection. The fabrication processes of the sensor are exhibited in Figure 7f including three main steps. The optic image shows the size can be adjusted. The SEM image shows the inside porous structure. By regulating the density and size of the sensing materials, the internal pore size of the three-dimensional graphene foam can be controlled. As shown in Figure 7g, this 3D structure graphene-based strain and pressure sensor exhibits good linearity over different strain ranges owing to the 3D structure. Figure 7h shows high sensitivity up to 0.36 kPa^−1^ in the range of 0–4 kPa under pressure, which demonstrates this 3D structure graphene-based strain and pressure sensor can both respond to pressure and tension.

The 3D structure is an important part of graphene-based pressure and strain sensors which can be mass produced in large quantities. The graphene 3D structure has both flexible and compressible and good mechanical repeatability of graphene. The 3D structure is a newly-developing form of graphene-based pressure and strain sensors, which also can be used in all transduction methods. The 3D structure graphene-based pressure and strain sensors can be pressured or stretched in all directions in an effective three-dimensional space. It can detect a large or tiny force and has negligible hysteresis, favorable linearity, and ultrahigh dynamic sensitivity. The 3D structure graphene-based pressure and strain sensors have great developing potential in the next decade.

## 4. Recent Development and Applications of Pressure Sensors

### 4.1. Wearable Devices in the Biomedical Field

Nowadays, developing wearable devices have drawn tremendous attention in improving health awareness of people. Compared with traditional medical diagnosis, E-skins possess the ability of human motion detection and health parameter collectivity and real-time monitoring. These detected signs can be broadly classified into the inner physiological signal, such as pulse and heart rate, along with the external motion and sound signals like gesture, gait state, and facial expressions [173,174,175,176]. Graphene-based sensors show great sensibility in pressure and strain detection, which have provided potential in remote medical diagnosis and in improvement of the bionic machine [177,178].

**Figure 8:**
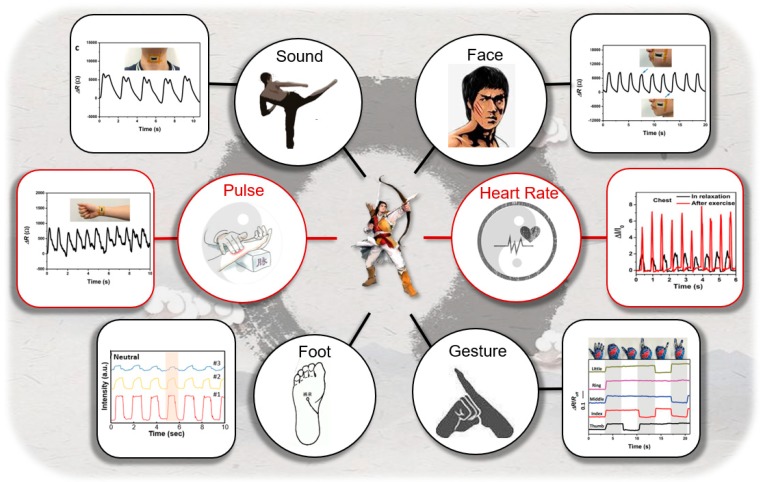
Monitoring in real-time of the graphene-based strain and pressure sensor for human motion detection [179,180,181]. The insets figures source from [185,186,187,188,189,190,191,192]. Copyright 2015, Wiley; Copyright 2017, Nature Publishing Group; Copyright 2018, American Chemical Society. (**a**) A graphene-based strain sensor applied to detect throat movement on human skin. (**b**) A graphene-based strain sensor applied to detect cheek movement on human skin. (**c**) A graphene-based strain sensor applied to detect wrist pulse on human skin. (**d**) A graphene-based pressure sensor applied to detect chest pulse on human skin. (**e**) A graphene-based pressure sensor applied to detect foot pressure on human skin. (**f**) A graphene-based strain sensor applied to distinguish six different hand positions.

The physiological signals like pulse and heart rate ought to be recorded with high-precision. Thus, detectability in the low-pressure region and low gauge factors and high sensitivity are essential to the reliable detection of the subtle pulse pressure located variously in the human body, mainly in the radial artery. Gong and co-workers proposed an efficient, low-cost, and ultrathin graphene-based strain sensor with high stretchability and sensitivity [179]. This sensor exhibits GF up to 9.9, stretchability reaches up to 350%, rapid response time about 22 ms, and repeatability greater than 5000 cycles. This graphene-based strain sensor has been applied in human motion detection, which can read radial artery pulse in real-time. In the experiment, the wrist pulses can be measured accurately under ordinary conditions (≈66 beats min^−1^), as demonstrated in Figure 8c. The same as with a typical radial artery pulse waveform, the curve obtained has two clearly distinguishable peaks, proving the high sensitivity of strain sensors. In light of the radial artery, the pulse wave originates from cardiovascular activity. A close association can be perceived between heart rate and pulse.

Shen and co-workers demonstrated a stable and highly sensitive graphene-based pressure sensor can be applied in human motion detection [180]. The pressure signals sensitivity up to 31.6 kPa^−1^ can be effectively and independently detected in this sensor, which makes it become a simply integrated sensor array with outstanding properties. As shown in the Figure 8d, the sensor records the current signal generated by the undulation of the chest during normal and exercise conditions within 6 s, which can realize the function of monitoring heartbeat signal in real time.

In the case of external motion and sound detection, regular monitoring of these signals is probably an efficient method to supervise the human kinematic state, which puts forward the requirements for sensitivity and detection range. Ren and co-workers proposed a highly sensitive and integrable graphene-based pressure sensor to detect dynamic gait motion [181]. This sensor has ultrahigh sensitivity up to 25.1 kPa^−1^ in a linearity range of 0−2.6 kPa, which demonstrates the ability to detect real-time human motion. Three pressure sensors were immobilized on the calcaneus, first metatarsal, and fifth metatarsal to monitor dynamic foot pressure on human skin. As shown in Figure 8e, this sensor can distinguish the neutral gait, supination gait, and pronation gait by monitoring changes in plantar pressure. Except for gait state, gesture and facial expressions are also required to be detected precisely, because of the relevant multiple joints of a single gesture and a large number of muscles on the face. It is worth mentioning the fist–palm salute, a specific gesture. To detect a gesture like that, Gong and co-workers integrated graphene-based strain sensors with existing clothing. As shown in Figure 8f, five strip sensors are stitched onto the finger surface of the glove to detect the movement of each finger, and the sensor assemblies were shown to have quick responses to finger motion. They also applied the sensors to detect facial expressions. Illustrated in Figure 8b, human facial movement can be clearly detected in a highly reproducible manner with a high signal to noise ratio. Another exigent function of e-skin is the identification of sound. The complex motion of the skin extending down a person’s throat while saying “Hello” can be reliably identified repeatable. As shown in Figure 8a, the two syllables of the word “Hello” correspond to the two small peaks in the output curve.

All in all, graphene-based strain and pressure sensors exhibit outstanding behavior and great potential for human motion detection and health monitoring, both for physiological and physical aspects. With improvement to the properties of the sensor, more accurate health monitoring and motion detection can be achieved [182,183,184].

### 4.2. From Smart Sensors to Potential Artificial Intelligence Sensors

In 1950, Alan Turing put forward the famous Turing test in “Can the machine think?” and “Computers and Intelligence”. Since then, the academic community has started to discuss the issue of machine thinking. The Dartmouth meeting in 1956 marked the birth of the concept of “artificial intelligence”. At present, artificial intelligence is gradually entering the commercial application stage and the critical point of the outbreak is being ushered in. Artificial intelligence is fully entering and reshaping human production and living space. From intelligent robots, smart homes to unmanned vehicles and unmanned factories, artificial intelligence technology is being widely used in various fields of social life and production, changing or even subverting our traditional cognition to the future of agriculture, manufacturing, and law enforcement [193]. The rapid development of artificial intelligence has had a great impact on working forms such as journalism, transportation, medicine, and sports [78]. With the integration of informationalization and industrialization, the boom of intelligence industries represented by robotics, mechatronics, automation, and human-machine interaction has become an important symbol of contemporary technological innovation [75,194]. In this section, we intend to use the studies of smart devices and artificial intelligence to point out that flexible sensors have great potential applications of artificial intelligence in the future.

**Figure 9:**
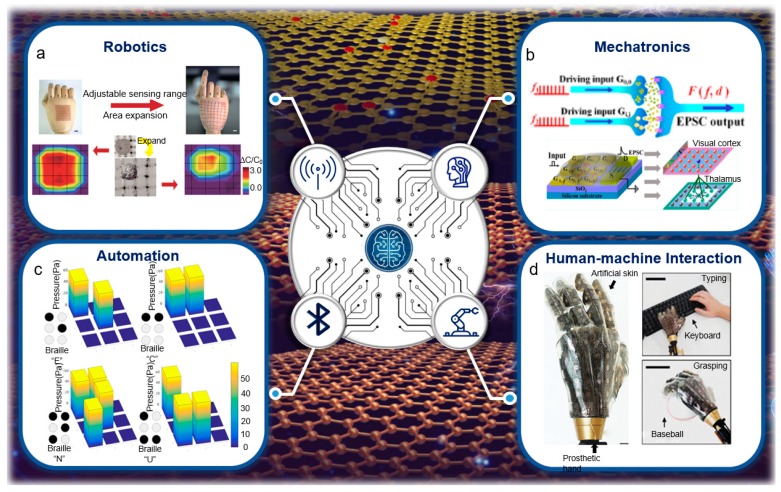
(**a**) A smart electronic skin on a hand, showing robotics [195]. Copyright 2018, Nature Publishing Group. (**b**) Mechatronics in artificial intelligence [196]. Copyright 2018, American Chemical Society. (**c**) Interaction demonstration of smart recognition of the Braille diagram. The Braille diagram is shown on the left corner. Pressure distribution of braille ‘E’ ‘C’ ‘N’ ‘U’ of the Gr-GO heterostructure film pressure sensor array is shown on the right. The pressure array wirelessly communicates with external devices via Bluetooth. (**d**) A smart electronic prosthetic hand, which can be used in human-machine interaction [197]. Copyright 2014, Nature Publishing Group.

Wang et al. presented a skin-inspired highly stretchable and conformable matrix network (SCMN) which expanded the application of wearable devices in the field of artificial intelligence [195]. The SCMN has multiple functions including detecting strain, pressure, temperature, light, humidity, magnetic field, and proximity. Figure 9a shows the real-time detection of pressure distribution and temperature distribution in the array. Figure 9a demonstrates its use in real-time spatial pressure mapping and temperature estimation. The spatial pressure mapping is implemented before and after the 300% expansion, which indicates that the SCMN is used to identify the location of the pressure load and estimate the size of the loaded object even when the network is stretched and expanded. In addition, this feature can be applied not only to determine the detection range, but also to define the detection area of other external stimuli. By adjusting the sensory nodes of the SCMN, it can be used for multi-function detection, which can be applied to human skin wearable devices in various test environments. Combined with artificial intelligence, the SCMN has a wider range of applications in human-machine interfaces, health monitoring technology, and biomimetic devices.

Wan et al. purposed a two-dimensional electrical double-layer transistor that successfully simulates basic neuromorphic behaviors such as excitatory postsynaptic currents and paired-pulse boosting [196]. The artificial vision neural network system was experimentally verified in these devices, which has great application potential in artificial intelligence and neuronal morphology. Figure 9b shows a simplified schematic of a vision system showing a clear three-layer abstract feedforward mode. The retina is the first layer and is responsible for receiving input. The thalamus is the second layer, a place where there is a one-to-one mapping between the retina and thalamic LGN cells. The visual cortex is the third layer, which is a place to create a many-to-one mapping from the second layer of cells to the third layer of cells. In general, this study indicates the future development of biomimetic nanotechnology.

Recently, our group proposed an ultrasensitive graphene heterostructure pressure sensor, which has not been published yet. Beyond the sensitivity of human skin and muscle, this graphene heterostructure pressure sensor has a pressure sensitivity of 5.64 kPa^−1^ and a simultaneous response frequency of at 10 kHz. We demonstrate that such multiple functional flexible arrays could be applied to automation with advanced artificial perception. For the interaction demonstration, an artificial recognition of the braille diagram is shown in Figure 9c. The spatial pressure distribution of braille ‘E’ ‘C’ ‘N’ ‘U’ exhibits that the pressure sensor could assist the blind to communicate with normal people. The pressure array wirelessly communicates with external portable devices via Bluetooth.

Kim et al. demonstrated a stretchable array of multi-kinetic smart prosthetic skin, which can perform multi-function measurements such as strain, pressure, humidity, and temperature [197]. This range of retractable sensors and actuators can respond to external stimuli and promote highly localized mechanical and thermal skin sensing, which provides a new direction for the artificial perception of prosthetic skin. Figure 9d shows an image of an artificial skin with integrated electronics laminated on a fake watch face. The artificial skin surface of the prosthesis is highly compliant and mechanically coupled to the curved surface of the prosthesis. The resistance changes in response to keyboard tapping and catching are monitored to explore the performance of the pressure response. The pressure sensor shows a fast and reliable response to external stimuli in both cases, which can be used in future artificial perception prosthetics.

In fact, there are still many challenges in implementing artificial intelligence applications for graphene-based strain and pressure sensors. For instance, how to record a large amount of measurement data and utilize them with artificial intelligence such as deep learning is a key to the application of graphene-based strain and pressure sensors. How to choose the appropriate model algorithm to calculate the parameters is also a difficult point. Furthermore, there are a handful of applications of graphene-based strain and pressure sensors in use, and we need to consider how to appropriately combine the graphene-based strain and pressure sensors with artificial intelligence.

## 5. Conclusions and Perspectives

Due to the magnificent properties including the mechanical, heat resisting, electrical conductivity, and flexibility of graphene, there is currently enormous research into graphene-based mechanical sensors. Graphene-based strain and pressure sensors are widely used in various emerging fields such as device system integration, health monitoring, human motion detection, human-machine interaction, and artificial perception. It is necessary to develop graphene-based strain and pressure sensors to discover potential applications especially in the prevailing trend of artificial intelligence.

In this review, we comprehensively describe ultramodern progress in graphene-based strain and pressure sensors including the sensing mechanism of diverse functional sensors, the main parameters of graphene-based strain and pressure sensors, graphene-based strain and pressure sensors in different dimensionality structures, as well as the potential applications of graphene-based strain and pressure sensors. A mass of publications and reports on the subject of graphene-based strain and pressure sensors demonstrate the urgent demands for various applications in the future.

Although tremendous progress has been achieved during the last decade in graphene-based strain and pressure sensors, there are also enormous challenges such as the negligible but present hysteresis, the balance between high sensitivity and large detection range, the high-frequency vibration test, and biological degradability which remain to be overcome. The large-area device system integration is also a challengeable project. Nowadays, sensor applications in emerging fields have become more and more miniaturized, integrated, and arrayed. This is an inevitable trend in the development of technology, which requires research to further combine sensors with integrated circuits. Additionally, although abundant ultra-sensitive sensors have been reported, novel materials and new-type sensing mechanisms still should be continuously optimized to meet the increasingly demanding application requirements. Moreover, emerging medical technologies such as real-time human health monitoring, prosthetic technology, and clinical medicine are urgently needed for artificial intelligence sensors for health monitoring. With the further development of information transmission technology, future mechanical sensors can be more intelligently controlled in different external environments, which are closer to real human skin functions. In the future, most of the mechanical sensors will have to face harsh operating conditions, which require high stability, environmental interference, and adaptive, self-compensating adjustment capabilities. At the same time, in order to ensure that electronic components and modules can achieve large-scale production, the cost also needs to be reduced. We need to improve performance in terms of both technology and cost. Overall, graphene-based strain and pressure sensors have a bright research potential and wide applicability.

## Figures and Tables

**Table 1:** Different parameters of the graphene-based strain sensors.

Sensing Materials	Transduction Mechanisms	Gauge Factor	Detection Range	Response Time	Reference
0D–1D–2D nanocomposite	0D Resistive Strain	2392.9 (ɛ = 62%)	0–62%	—	[136]
PDCY–rGO	1D Resistive Strain	35 (ɛ = 0.2%)	0.2–100%	<100 ms	[137]
CSF	1D Resistive Strain	37.5 (ɛ = 250–500%)	0–500%	<70 ms	[138]
AgNW/Graphene	1D Capacitance Strain	—	5–200%	<1 ms	[139]
PMSCSS	2D Resistive Strain	647 (ɛ = 0.14%)	0–0.22%	0.625 ms	[140]
GWF	2D Resistive Strain	500 (ɛ = 2%)	0–40%	<30 ms	[141]
BGF/BGFM	3D Resistive Strain	6.5 (ɛ = 10%)	0–50%	—	[142]
Graphene/PDMS	3D Resistive Strain	55.1 (ɛ = 25%)	0–64%	400 ms	[143]
Graphene	1D Resistive Strain	42.8 (ɛ = 9%)	0–24%	1.1 s	[144]
Graphene	2D Resistive Strain	1037 (ɛ = 2%)	0–4%	—	[145]

**Table 2:** Different parameters of the graphene-based pressure sensors.

Sensing Materials	Transduction Mechanisms	Sensitivity	Detection Range	Response Time	Reference
PTNWs/Graphene	1D Piezoelectric Pressure	9.4 × 10^−3^ kPa^−1^	0–1.5 kPa	5–7 ms	[34]
rGO/PVDF	1D Resistive Pressure	15.6 kPa^−1^	1.2 Pa–60 kPa	5 ms	[146]
rGO	2D Capacitance Pressure	0.8 kPa^−1^	0.24 Pa–4 kPa	<100 ms	[111]
LSG	2D Resistive Pressure	0.96 kPa^−1^	10–100 kPa	0.4 ms	[147]
ACNT/Graphene	2D Resistive Pressure	19.8 kPa^−1^	0.6 Pa–0.3 kPa	<16.7 ms	[148]
WG	2D Resistive Pressure	6.92 kPa^−1^	0–5 kPa	—	[149]
Graphene hybrid	2D Resistive Pressure	0.032 kPa^−1^	0–100 kPa	10 kHz	[150]
MX/rGO	3D Resistive Pressure	22.56 kPa^−1^	0–3.5 kPa	<200 ms	[151]
rGO/PU	3D Resistive Pressure	0.26 kPa^−1^	0–10 kPa	—	[86]
G-S	3D Capacitance Pressure	1.04 kPa^−1^	0–20 kPa	<5 ms	[152]
rGO/PI/HT	3D Resistive Pressure	0.36 kPa^−1^	0.2 Pa–14 kPa	<80 ms	[153]
OPG	3D Resistive Pressure	313.23 kPa^−1^	0–4 kPa	28 ms	[154]
Graphene	2D Resistive Pressure	17.2 kPa^−1^	0–20 kPa	—	[155]
Graphene	2D Capacitance Pressure	0.33 kPa^−1^	0–5 kPa	<20 ms	[112]
Graphene	3D Resistive Pressure	110 kPa^−1^	0.2 Pa–75kPa	<30 ms	[156]
